# Genomic and Computational Analysis of Novel SNPs in *TNP1* Gene Promoter Region of *Bos indicus* Breeding Bulls

**DOI:** 10.1155/2022/9452234

**Published:** 2022-03-15

**Authors:** Kashif Hameed Anjum, Asif Nadeem, Maryam Javed, Hafiz Ishfaq Ahmad, Amjad Riaz, Wasim Shehzad, Jahanzaib Azhar, Muhammad Fahad Bhutta

**Affiliations:** ^1^Institute of Biochemistry & Biotechnology, University of Veterinary and Animal Sciences, Lahore, Pakistan; ^2^Department of Biotechnology, Virtual University of Pakistan, Lahore, Pakistan; ^3^Department of Animal Breeding and Genetics, University of Veterinary and Animal Sciences, Lahore, Pakistan; ^4^Department of Theriogenology, University of Veterinary and Animal Sciences, Lahore, Pakistan; ^5^Semen Production Unit, Qadirabad, Sahiwal, Pakistan

## Abstract

Transition nuclear proteins (TNPs), the principal proteins identified in the condensing spermatids chromatin, have been found to play a key role in histone displacement and chromatin condensation during mammalian spermatogenesis. One such gene belonging to the TNP family called *TNP1* gene is abundantly expressed in the regulation of spermatogenesis, and its sequence is remarkably well conserved among mammals. Genomic analysis, by sequencing and computational approach, was used to identify the novel polymorphisms and to evaluate the molecular regulation of *TNP1* gene expression in Sahiwal cattle breeding bulls. DNA samples were sequenced to identify novel single nucleotide polymorphisms (SNPs) in the *TNP1* gene. Modern computational tools were used to predict putative transcription factor binding in the *TNP1* promoter and CpG islands in the *TNP1* promoter region. In the *TNP1* gene, four SNPs, three TATA boxes, and one CAAT box were identified. One CAAT box was discovered at 89 bp upstream of start site ATG. The computational analyses indicated that the polymorphisms inside the promoter sequence results in an added HNF-1 transcription factor binding site. In contrast, the other variations may remove the naturally occurring SRF transcription factor binding site. The CpG islands in the *TNP1* promoter region were predicted to be absent by the MethPrimer program before and after SNP site mutations. These findings pave the way for more research into the *TNP1* gene's promoter activity and the links between these SNPs and reproductive attributes in the Sahiwal breeding bulls.

## 1. Introduction

Bulls have gained major importance in animal breeding since a specific bull can mate numerous females. A lack of breeding competence in the bull can have a greater influence on the productivity of a herd than female reproductive issues. Bull genomics is considered the primary material through which genetic improvements can efficiently be accomplished. Some in fertility and subfertility problems have been observed for the culling of Murrah, Sahiwal, and KF bulls. One of the significant causes of infertility and subfertility is spermatozoa maturation [1]. Anomalies in the DNA/RNA or protein can lead to defective spermatozoa, which can no longer fuse functionally with the oocyte. Such events cannot progress towards induction of embryonic development, resulting in infertility [2–4]. Spermatozoa maturation has been linked to the DNA stability, nuclear vacuoles quality, and chromatin organization [5, 6]. The sperm nucleus experiences substantial reorganization, with histones being replaced by numerous nuclear proteins. TNPs are the predominant proteins identified in the condensed spermatids chromatin and are critical for chromatin condensation and histone replacement during spermatogenesis in animals. The *TNP1* gene in *Bos taurus* is found on chromosome 2q42-q43 [7] and encodes a 6.2 kDa protein with 55 residues (https://www.ncbi.nlm.nih.gov/protein/XP019841056.1) with approximately 20% lysine and 20% arginine spread equally [8]. *TNP1* is an extensively produced protein during spermatogenesis regulation, and its sequence is substantially similar among mammals [9]. Polymorphisms in the human *TNP1* gene appear to be linked to DNA degradation in individuals with azoospermia and varicocele [10].

Similarly, the lack of both *TNP1* and *TNP2* renders the mouse model sterile, whereas subfertile null mice mutants lack *TNP1* and/or *TNP2* [11–13]. Moreover, a study also reported that three single nucleotide polymorphisms are present in the *TNP1* gene at locations 205, 340, and 346 bp, and all these SNPs are located in the intronic regions. When the influence of this polymorphism on mass activity, individual motility, and spermatozoa maturation was investigated, it was found to have a substantial impact on spermatozoa development but was nonsignificant on mass activity and sperm motility [14–16].

In current study, SNPs of the *TNP1* gene in breeding bulls were identified using a DNA sequencing technique. Furthermore, computational tools were utilized for promotor prediction, prediction of transcription factor (TF) binding sites and CpG islands, and the influence of discovered SNPs on the CpG Islands. The primary purpose of the present study includes creating a foundation to confirm the effect of SNPs on *TNP1* gene expression and the relationship between reduced spermatogenesis in breeding bulls and the SNPs.

## 2. Materials and Methods

### 2.1. Experimental Animals

Semen straw samples (*n* = 50) of Sahiwal cattle breeding bulls were collected from Semen Production Unit (SPU), Qadirabad, Sahiwal, Pakistan. Semen samples of bulls were transported in liquid nitrogen and stored temporarily in the freezer at −20°C before DNA extraction.

### 2.2. Genomic DNA Extraction

Extraction and purification of genomic DNA from semen straws were conducted using the phenol-chloroform method described [17]. Agarose gel electrophoresis was used to check the quality of extracted DNA. Furthermore, the Nanodrop (Thermo Fisher Scientific) spectrophotometer was also used to determine the concentration of DNA based on optical density at 260 and 280 nm. DNA samples having the OD_260_/OD_280_ ratio of 1.83 ± 0.005 were used for further work.

### 2.3. PCR Conditions and Amplification

Primer3 software was used to create gene-specific oligonucleotide primers (http://frodo.wi.mit.edu/cgi-bin/primer3/primer3http://www.slow.cgi) of the *TNP1* bovine gene (NCBI Reference Sequence: NC_032651.1) (Supplementary Table 1). The total volume of the polymerase chain reaction was 25 *μ*L, which included 2 *μ*L template DNA (100 ng/*μ*L), 2.5 *μ*L 10x PCR buffer with 2.5 *μ*L MgCl_2_, 1 *μ*L of each forward and reverse primer (both at 10 mol/L), 2.5 *μ*L of dNTPs, 0.5 *μ*L of 5U of Taq DNA polymerase, and 13 *μ*L of nuclease-free water (Thermo Fisher Scientific). The PCR amplification program was as follows: initial denaturation step at 94°C for 3 minutes, followed by 35 cycles of denaturation step at 94°C for 30 seconds, annealing step at 58°C for primer set for 30 seconds, and extension at 72°C for 1 minute, and final extension step at 72°C for 10 minutes. To store PCR products, −20°C refrigeration temperature was used.

### 2.4. Sequencing and SNPs Analysis

Gel electrophoresis was used to evaluate the quality and size of amplified PCR products on 1.2 percent agarose gel. Ethanol was used for the precipitation of PCR products. 40 *μ*L of 75% ethanol was added to each 10 *μ*L reaction to a final concentration of 60%. The reaction mixtures were vortexed and left at room temperature for 20 min. Then, these were centrifuged at 16000 x g (14000 rpm for 20 min) at 4°C. The supernatant was discarded, and pellets were washed with 100 *μ*L of 70% ethanol. Then, pellets were dissolved in 15 *μ*L of deionized water. After sequencing PCR, precipitated PCR products were sequenced using dye-labeled dideoxy terminator sequencing using ABI Genetic Analyzer 3130 XL (Applied Biosystem Inc., Foster City, CA, USA). The sequencing results of the samples were aligned by BLAST and ClustalW with the *TNP1* reference gene sequence of *Bos indicus* (NCBI reference sequence: NC_032651.1). The background noise in the amplified PCR product sequence chromatogram was trimmed using 4Peaks software (https://nucleobytes.com/) from both sides and pairwise alignment was obtained with NCBI ref. seq. accession. No. NC_032651.1 using NCBI online BLAST software. MEGA6 [18, 19] was utilized to analyze SNPs in the *TNP1* gene amplified from breeding bulls.

### 2.5. Computational Analysis of TNP1 Gene from Breeding Bulls

Different functional elements of *TNP1* gene, including promoter region, CpG islands, and transcription factor binding sites, were predicted and analyzed through different computational tools.

#### 2.5.1. Promoter Region Prediction

The promoter is the central DNA element separated into distal, proximal, and core regions and is meant to regulate the transcription of genes. To infer the promoter areas of *TNP1*, three distinct computational tools were used in this investigation, including Promoter Scan [20], Promoter 2.0 Prediction Server [21, 22], and Neural Network Promoter Prediction [23]. Promoter Scan is the computer program meant to recognize the high percentage of Pol II promoter sequences while allowing very few false positives. Similarly, Promoter 2.0 and Neural Network Promoter Prediction server predict the transcription start sites of vertebrates Pol II promoters based on neural network principles and genetic algorithm.

#### 2.5.2. Prediction of TF Binding Sites

A computational algorithm called AliBaba2.1 [24] was used to assess the *TNP1* promoter's binding sites of the transcription factor in breeding bulls. AliBaba2.1 server is for context-specific identification of transcription factor binding sites, and prediction is based on constructing matrices on the fly from TRANSFAC 4.0 sites [25].

#### 2.5.3. Prediction of CpG Islands

CpG islands are those regions in DNA where GC content is high. They are prominently found within promoter region or in the vicinity of promoter region which act as markers for the regulation of gene expression when undergoing methylation. MethPrimer is a computer program based on Primer3, and it is specifically developed for designing primers for methylation mapping. It works by taking the DNA sequence as input and then searching the sequence for potential CpG islands [26]. In the present study, MethPrimer (http://itsa.ucsf.edu/∼urolab/methprimer) was utilized to predict the CpG islands present in the *TNP1* gene's promoter.

#### 2.5.4. Analysis of the Genetic Variation

All allelic and genotypic frequencies for newly found SNPs were quantified and tested in the current investigation using the earlier developed method [27].

## 3. Results and Discussion

*TNP1* belongs to a class of TNP genes important in histone-to-protamine replacement during spermatid nuclear transformation. This gene family is believed to alter sperm nuclear chromatin to ensure stability after transformation to a DNA-protamine complex [28]. Suppression of the *TNP1* gene expression causes failure of spermatogenesis to occur, resulting in a round spermatid arrest [29]. Moreover, polymorphisms in the *TNP1* gene have been shown to impair chromatin structure of sperms, ultimately leading to diminished fertility in mice [13], as well as significantly affecting spermatozoal maturation negatively in humans [30] and the Murrah buffalo bulls [14]. A broad picture of how SNPs in *TNP1* gene in breeding bulls affect their reproductive abilities is still lacking. Moreover, there are no studies on the *TNP1* gene SNPs on the local breeding bull population in Pakistan. So, in the current study, the SNPs in the promotor region of *TNP1* gene, which can primarily lead to reduced spermatogenesis in the Sahiwal breeding bulls (a local bull breed) is investigated.

**Figure 1:**
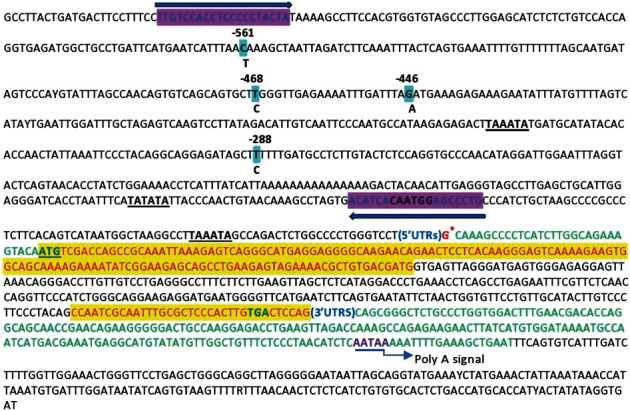
*TNP1* genomic DNA sequences and primers for PCR amplification and sequencing. The recognition site nucleotide sequences, i.e., TATA box nucleotide, are highlighted. Shadowed letters represent single SNPs, while minor alleles are noted beneath the nucleotide. The transcription start site is shown with an asterisk. The arrows above and below the DNA sequence represent the primers employed for polyA-additional signal PCR amplification (the polyA signal from the straight arrow is added to the *TNP1* mRNA). Yellow areas represent axons in the TNP1 gene.

We found four SNPs in the promoter region at −568 nt, −468 nt, −446 nt, and −288 nt from transcription start site upstream, while positions of these SNPs in BLAST pairwise alignment were 75C > T, 161T >C, 183G > A, and 341T > C (Figure 1). Three TATA boxes and one CAAT box were identified in the studied fragment (Table 1). Transcription initiation sites like TATA signal were present at positions 25 to 30, 122 to 127, 339 to 344, and one CAAT box at 84–89. All the sites identified were located in the negative strand (−) with six nucleotides each. These findings are consistent with Ranjan et al., who reported results of three TATA boxes in the *TNP1* promotor region and disagreement with them concerning the position of TATA boxes [31]. We identified one CAAT box at position 89 bp upstream of ATG while Ranjan et al. found two CATT boxes at positions 60 and 121 bp upstream of ATG [31].

### 3.1. Single Nucleotide Polymorphisms Predict Putative Transcription Factor Binding in the *TNP1* Promoter

The sites where DNA sequences are with transcription factors are called binding sites. Alterations in transcription factor binding sites could cause a significant impact on transcription factor binding to gene regulatory areas and gene expression products [27]. We examined the region and discovered SNPs that could change transcription factor binding sites. According to the current research findings, the SNP is identified at the promoter area locus, leading to an additional HNF-1 transcription factor binding site. In contrast, another SNP may result in the loss of the original SRF transcription factor binding site. SRF interacts with various other proteins and binds to DNA to regulate the transcription of genes [32, 33]. As a result, it is suggested that these SNP locations may influence the expression of *TNP1* gene via changes in transcription factors.

RNA polymerase recognizes and binds DNA at the promoter region. DNA sequence changes in this area may alter binding sites of the transcription factor to influence gene expression.  Table 2 shows the transcription factor alterations indicated by the online computational tool, AliBaba2.1, in the *TNP1* promoter region. As per the findings, a C ⟶ T polymorphism happened in the gene's promoter region, culminating in an extra binding site for the HNF-1 transcription factor. Another polymorphism, i.e., T ⟶ C at the locus may remove the initial binding site of SRF transcription. The G ⟶ A variation can also result in the loss of RAP transcription factor binding sites, while the T ⟶ C polymorphism could remove the original C/EBPalp binding site. As a result, such mutations, especially at recently found loci, can potentially eliminate the original transcription factor binding sites while generating new transcription factor binding sites, changing the control of *TNP1* expression in cattle.

### 3.2. CpG Islands in the Promoter Region Prediction Results

CpG islands are unmethylated gene sequences that can be coupled with particular transcription factors to enable regular expression [34]. When CpG islands are methylated, the transcription of the relevant genes is repressed, changing gene expression [35]. CpG islands are prone to methylation, decreasing transcription factor binding, and influencing gene expression.

**Figure 2:**
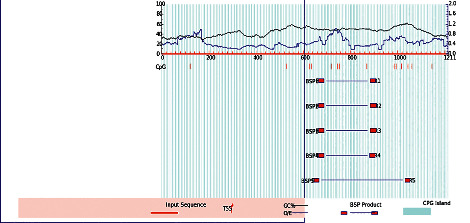
CpG island prediction using MethPrimer software.

To determine the presence of CpG islands in the 1211-bp sequence of the *TNP1* promoter area before and after SNP site mutations, we used MethPrimer software. This was done to investigate the influence of such SNPs on modifications of the anticipated CpG island in the promoter region of *TNP1*. The MethPrimer software (default parameter values were chosen for the CpG island length >100 bp, CG percent >50%, and Obs/Exp >0.6) projected no CpG islands in the *TNP1* gene promoter area (Figure 2).

## 4. Conclusion

In the present investigation, the effect of SNPs on *TNP1* gene expression of the local (Sahiwal) breeding bulls was examined. Genomic sequencing analyses of the gene and its various important sites identified four SNPs, three TATA boxes, and one CAAT 89 bp upstream of the ATG start codon. The identified SNP and variations in other genetic elements are expected to be correlated with altered spermatozoa maturation. In the *TNP1* promoter region, a SNP resulted in an added HNF-1 transcription factor binding site, which can alter the *TNP1* expression controlling genetic apparatus. Moreover, the absence of CpG islands in the promoter region of *TNP1* was also observed, which is expected for transcription of the *TNP1* gene significantly. The present study's findings could help us understand the role of various genetic sequences and how their variations affect the expression of *TNP1* gene in Sahiwal breeding bulls. However, the limited size of the sample population is a hindrance to forming a definite conclusion. So, in the future, a larger sample size is required to establish a causal role of the *TNP1* gene control on reproductive attributes of breeding bulls, the predictions made in the study form the groundwork for future research.

## Figures and Tables

**Table 1:** Sites found in the TNP1 gene promoter region from the transcription start point.

Site	Location	Strand	Size	Sequence
TATA	339–344	—	6	TAAATA
	122–127	—	6	TATATA
	25–30	—	6	TAAATA

CAAT	84–89	—	6	CAATGG

**Table 2:** Variations in transcription factors before and after a SNP in the *TNP1* promoter region.

SNPs site	Base variations	Transcription factor	Base sequence of transcription factor binding site
109072682	C	—	—
	T	HNF-1	cagctaataa

109072589	T	SRF	catggagcca
	C	—	catggagcca

109072567	G	RAP	gtgtctg
	A	—	—

109072409	T	C/EBPalp	tatttatttt
	C	—	tatttatttt

## Data Availability

The data will be openly available to all readers.

## Supplementary Materials

Supplementary Materials List of primers used in the study, genotype frequency, and CpG island prediction in exonic and intronic regions of TPN1 gene. Table S-1: list of primers of TNP1 gene. Table S-2: allelic and genotypic frequency of identified polymorphisms. Figure S-1: the CpG island prediction in exonic and intronic regions of TPN1 gene.
