# Supplementary material for: Genomic and Computational Analysis of Novel SNPs in TNP1 Gene Promoter Region of Bos indicus Breeding Bulls
Source: Genet Res (Camb). 2022 Mar 15;2022:9452234. doi: 10.1155/2022/9452234 (PMC8941572; doi:10.1155/2022/9452234)
Supplement: Supplementary Materials — List of primers used in the study, genotype frequency, and CpG island prediction in exonic and intronic regions of TPN1 gene. Table S-1: list of primers of TNP1 gene. Table S-2: allelic and genotypic frequency of identified polymorphisms. Figure S-1: the CpG island prediction in exonic and intronic regions of TPN1 gene. [file 9452234.f1.docx]

**Supplementary Data**

**List of primers used in the study, genotype frequency and CpG island prediction in exonic and intronic regions of TPN1 gene.**

**Table S-1: List of Primers of *TNP1* gene**

| Sr. No. | Primer Name | 5’-3’ Sequence | Amplified PCR Product Size  (bp) | Tm  (°C) |
| --- | --- | --- | --- | --- |
| 1 | TNP1 F1 | TTGTCCACCTCCCCCTACTA | 588 | 59.40 |
| 2 | TNP1R1 | CAGGGCTCCATTGTGATGT |  | 59.49 |
| 3 | TNP1 F2 | CAGGTGCCCAACATAGGATT | 569 | 59.81 |
| 4 | TNP1 R2 | AGATGGGAACCTGGTTGAGA |  | 59.51 |
| 5 | TNP1 F3 | AAGAGGATGAATGGGGTTCA | 489 | 59.34 |
| 6 | TNP1 R3 | GGTCAGAGTGCACACAGATGA |  | 59.89 |

**Table S-2: Allelic and genotypic frequency of identified polymorphisms**

| **Sr. No.** | **SNP ID** | **Allele Frequency** | | **Genotype Frequency** | | |
| --- | --- | --- | --- | --- | --- | --- |
|  |  | A | B | AA | AB | BB |
| 1 | TN1 | 0.77 | 0.23 | 0.74 | 0.06 | 0.20 |
| 2 | TN2 | 0.64 | 0.36 | 0.60 | 0.08 | 0.32 |
| 3 | TN3 | 0.67 | 0.33 | 0.64 | 0.06 | 0.30 |
| 4 | TN4 | 0.71 | 0.29 | 0.66 | 0.10 | 0.24 |

Figure S-1: The CpG island prediction in exonic and intronic regions of TPN1 gene


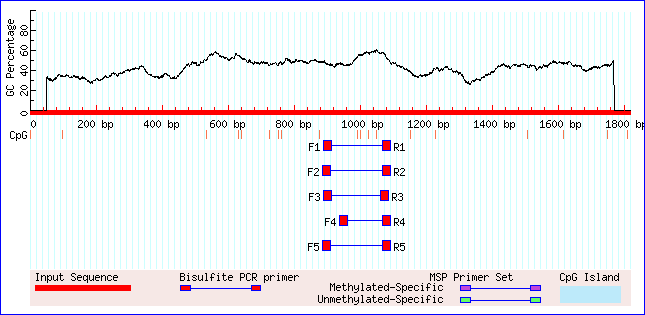


Table S-3:Molecular evolutionary genetics analysis

Sequence type explicitly set to DNA

Sequence format is Pearson

Sequence 1: NC_032651.1 456 bp

Sequence 2: PAKSAHIWAL 439 bp

Sequence 3: XM_005889637.2 324 bp

Sequence 4: BK006511.1 206 bp

Start of Pairwise alignments

Aligning...

Sequences (1:2) Aligned. Score: 98

Sequences (1:3) Aligned. Score: 98

Sequences (1:4) Aligned. Score: 100

Sequences (2:3) Aligned. Score: 98

Sequences (2:4) Aligned. Score: 99

Sequences (3:4) Aligned. Score: 99

Guide tree file created: [[clustalw.dnd]](https://www.genome.jp/tools-bin/pushfile?220202065047XHlDa+clustalw.dnd)

There are 3 groups

Start of Multiple Alignment

Aligning...

Group 1: Sequences: 2 Score:3914

Group 2: Sequences: 3 Score:6094

Group 3: Sequences: 4 Score:5326

Alignment Score 11000

CLUSTAL-Alignment file created [[clustalw.aln]](https://www.genome.jp/tools-bin/pushfile?220202065047XHlDa+clustalw.aln)

[clustalw.aln](https://www.genome.jp/tools-bin/pushfile?220202065047XHlDa+clustalw.aln)

CLUSTAL 2.1 multiple sequence alignment

NC_032651.1 CCTTGGAGCATCTCTCTGTCCACCAGGTGAGATGGCTGCCTGATTCATGAATCATTTAAC

BK006511.1 ------------------------------------------------------------

PAKSAHIWAL CCTTGGAGCATCTCTCTGTCCACCAGGTGAGATGGCTGCCTGATTCATGAATCATTTAAT

XM_005889637.2 ------------------------------------------------------------

NC_032651.1 AAAGCTAATTAGATCTTCAAATTTACTCAGTGAAATTTTGTTTTTTTAGCAATGATAGTC

BK006511.1 ------------------------------------------------------------

PAKSAHIWAL AAAGCTAATTAGATCTTCAAATTTACTCAGTGAAATTTTGTTTTTTTAGCAATGATAGTC

XM_005889637.2 -----------------------------------------------------GATAGTC

NC_032651.1 CCAYGTATTTAGCCAACAGTGTCAGCAGTGCTTGGGTTGAGAAAATTTGATTTAGATGAA

BK006511.1 ------------------------------------------------------------

PAKSAHIWAL CCATGTATTTAGCCAAAAGTGTCAGCAGTGCTCGGGTTGAGAAAATTTGATTTAAATGAA

XM_005889637.2 CCACGTATTTAGCCAACAGTGTCAGCAGTGCTCAGGTTGAGAAAATTTGATTTAGATGAA

NC_032651.1 AGAGAAAGAATATTTATGTTTTAGTCATAYTGAATTGGATTTGCTAGAGTCAAGTCCTTA

BK006511.1 -----------------------------------------------------GTCCTTA

PAKSAHIWAL AGAGAAAGAATATTTATGTTTTAGTCATATTGAATTGGATTTGCTAGAGTCAAGTCCTTA

XM_005889637.2 AGAGAAAGAATATTTATGTTTTAGTCATATTGAATTGGATTTGCTAGAGTCAAGTCCTTA

*******

NC_032651.1 TAGACATTGTCAATTCCCAATGCCATAAGAGAGACTTAAATATGATGCATATACACACCA

BK006511.1 TAGACATTGTCAATTCCCAATGCCATAAGAGAGACTTAAATATGATGCATATACACACCA

PAKSAHIWAL TAGACATTGTCAATTCCCAATGCCATAAGAGAGACTTAAATATGATGCATATACACACCA

XM_005889637.2 TAGACATTGTCAATTCCCAATGCCATAAGAGAGACTTAAATATGATGCATATACACACCA

************************************************************

NC_032651.1 ACTATTAAATTCCCTACAGGCAGGAGATAGCTTTTTTGATGCCTCTTGTACTCTCCAGGT

BK006511.1 ACTATTAAATTCCCTACAGGCAGGAGATAGCTTTTTTGATGCCTCTTGTACTCTCCAGGT

PAKSAHIWAL ACTATTAAATTCCCTACAGGCAGGAGATAGCTCTTTTGATGCCTCTTGTACTCTCCAGGT

XM_005889637.2 ACTATTAAATTCCCTACAGGCAGGA--TAGCTTTTTTGATGCCTCTTGTACTCTCCAGGT

************************* ***** ***************************

NC_032651.1 GCCCAACATAGGATTGGAATTTAGGTACTCAGTAACACCTATCTGGAAAACCTCATTTAT

BK006511.1 GCCCAACATAGGATTGGAATTTAGGTACTCAGTAACACCTATCTGGAAAACCTCATTTAT

PAKSAHIWAL GCCCAACATAGGATTGGAATTTAGGTACTCAGTAACACCTATCTGGAAAACCTCATTTAT

XM_005889637.2 GCCCAACATAGGATTGGAATTTAGGTACTCAGTAACACCTATCTGGAAAACCTCATTTAT

************************************************************

NC_032651.1 CATTAAAAAAAAAAAAAAAGACTACAACATTGAGGG

BK006511.1 CATTAAAAAAAAAAAAAAA-----------------

PAKSAHIWAL CATTAAAAAAAAAAAAAAA-----------------

XM_005889637.2 CATTAAAAAAAAAAAAAAA-----------------

*******************

[clustalw.dnd](https://www.genome.jp/tools-bin/pushfile?220202065047XHlDa+clustalw.dnd)

(NC_032651.1:0.00343,

(PAKSAHIWAL:0.00740,

XM_005889637.2:0.00803)

:0.00300,BK006511.1:-0.00343);
